# Anatomical Connectivity-Based Strategy for Targeting Transcranial Magnetic Stimulation as Antidepressant Therapy

**DOI:** 10.3389/fpsyt.2020.00236

**Published:** 2020-04-03

**Authors:** Qi Tao, Yongfeng Yang, Hongyan Yu, Lingzhong Fan, Shuxin Luan, Lei Zhang, Hua Zhao, Luxian Lv, Tianzi Jiang, Xueqin Song

**Affiliations:** ^1^Department of Psychiatry, The First Affiliated Hospital of Zhengzhou University, Zhengzhou, China; ^2^Biological Psychiatry International Joint Laboratory of Henan, Zhengzhou University, Zhengzhou, China; ^3^Henan Psychiatric Transformation Research Key Laboratory, Zhengzhou University, Zhengzhou, China; ^4^Academy of Medical Sciences, Zhengzhou University, Zhengzhou, China; ^5^Department of Psychiatry, Henan Mental Hospital, The Second Affiliated Hospital of Xinxiang Medical University, Xinxiang, China; ^6^Henan Key Lab of Biological Psychiatry, Xinxiang Medical University, Xinxiang, China; ^7^International Joint Research Laboratory for Psychiatry and Neuroscience of Henan, Xinxiang, China; ^8^Key Laboratory for NeuroInformation of Ministry of Education, School of Life Science and Technology, University of Electronic Science and Technology of China, Chengdu, China; ^9^Brainnetome Center, Institute of Automation, Chinese Academy of Sciences, Beijing, China; ^10^National Laboratory of Pattern Recognition, Institute of Automation, Chinese Academy of Sciences, Beijing, China; ^11^University of Chinese Academy of Sciences, Beijing, China; ^12^Department of Physiology, College of Basic Medical Sciences, Jilin University, Jilin, China; ^13^Department of Psychiatry, The First Bethune Hospital of Jilin University, Changchun, China

**Keywords:** treatment-resistant depression (TRD), transcranial magnetic stimulation (TMS), anatomical connectivity, subgenual anterior cingulated cortex (sACC), prefrontal cortex (PFC)

## Abstract

**Objectives:**

Abnormal activity of the subgenual anterior cingulate cortex (sACC) is implicated in depression, suggesting the sACC as a potentially effective target for therapeutic modulation in cases resistant to conventional treatments (treatment-resistant depression, TRD). We hypothesized that areas in the prefrontal cortex (PFC) with direct fiber connections to the sACC may be particularly effective sites for treatment using transcranial magnetic stimulation (TMS). The aim of this study was to identify PFC sites most strongly connected to the sACC.

**Methods:**

Two neuroimaging data sets were used to construct anatomic and functional connectivity maps using sACC as the seed region. Data set 1 included magnetic resonance (MR) images from 20 healthy controls and Data set 2 included MR images from 15 TRD patients and 15 additional healthy controls. PFC voxels with maximum values in the mean anatomic connection probability maps were identified as optimal sites for TMS.

**Results:**

Both right and left PFC contained sites strongly connected to the sACC, but the coordinates (in Montreal Neurological Institute space) of peak anatomic connectivity differed slightly between hemispheres. The left PFC site connected directly to the sACC both anatomically and functionally, while the right PFC site was functionally connected to the posterior cingulate cortex (PCC).

**Conclusions:**

Both left and right PFC are functionally connected to regions implicated in depression, the sACC and PCC, respectively. These bilateral PFC sites may be effective TMS targets to treat TRD.

## Introduction

Major depressive disorder (MDD) can be effectively treated in the majority of cases by medication, psychotherapy, or a combination of both, but more than one third of patients fail to respond to these standard interventions and other treatments, termed treatment-resistant depression or TRD cases (1). Chronic depression is associated with reduced productivity and quality of life as well as increased suicide risk, so alternative treatments for TRD are required.

Transcranial magnetic stimulation (TMS), deep brain stimulation (DBS), and vagus nerve stimulation (VNS) exert antidepressant effects by modulating activity within specific neural networks associated with emotional regulation and cognition (2–5). Vagus nerve stimulation has been approved by the United States Food and Drug Administration (FDA) for TRD treatment, although not for management of acute illness (6). Clinically significant antidepressive effects have also been observed following chronic DBS of the subcallosal cingulate white matter (4, 7) and subgenual anterior cingulate cortex (sACC) (8, 9) with good patient tolerability. However, DBS treatment is invasive and efficacy for TRD is still under investigation (10). Alternatively, TMS is non-invasive and well tolerated, with no evidence of cognitive impairment and few reports of medical complications. While TMS has been approved by the FDA for TRD, its effect size is generally modest compared to DBS (6). This lower efficacy may result from uncertainty regarding therapeutic mechanisms and the optimal regimens and target sites. For instance, while the prefrontal cortex (PFC) is widely regarded as an effective stimulation site, the precise subregions of left and right PFC evoking the optimal therapeutic response are unclear (11–13).

Depression involves dysfunction in a distributed network of cortical and limbic regions, including the ACC (2, 14–16). Previous studies have suggested that DBS of the sACC can effectively reverse symptoms of TRD (9), and is particularly effective against the associated cognitive deficits (3, 9, 15, 16). There is also evidence that functional connectivity of the sACC may predict treatment response to TMS (15, 16). An early review by Mayberg (3) suggested that normalization of sACC hyperactivity was a prerequisite for symptom remission (3). Indeed, reduced sACC activity has been reported following successful treatment with a variety of methods, including TMS (17, 18) and VNS (19). These studies suggest that the sACC acts as a hub within a critical depression-related circuit and that effects on activity within this circuit are strongly related to antidepressant efficacy.

The sACC and rostral ACC (rACC) differ in anatomic connectivity, cytology, and neurotransmitter receptor organization (20, 21). Further, studies comparing different left prefrontal cortex (PFC) stimulation sites found that antidepressant efficacy was related to the functional connectivity with deeper limbic regions, especially the sACC (22, 23). Fox and colleagues found that the sACC and dorsolateral PFC (DLPFC) are intrinsically anticorrelated, and suggested that the functional link between these two regions is strongly related to depression and treatment response (24). This anatomic connectivity with the sACC suggests that the PFC may be the optimal TMS site for neuromodulation. To our knowledge, most TMS studies for MDD have applied stimuli to the left dorsolateral PFC (DLPFC) (13, 25, 26). In present study, we identified PFC regions of strongest anatomic and functional connectivity to sACC as potential sites for optimal TMS treatment response.

## Materials and Methods

### Participants and Clinical Diagnosis

Two independent data sets were used in the present study. Data set 1 consisted of twenty healthy, right-handed subjects (10 males and 10 females, mean age=18.5 ± 1.5 years old) recruited by advertisement from the University of Electronic Science and Technology of China, Chengdu. None of the participants had a history of psychiatric or neurological diseases, and none had any contraindications for magnetic resonance imaging (MRI). All participants signed a consent form approved by the Medical Research Ethics Committee of the University of Electronic Science and Technology of China after a full explanation of study objectives and procedures. The Ethics Committee of the First Affiliated Hospital of Jilin University (China) approved the study protocol.

Data set 2 consisted of fifteen right-handed TRD patients (6 males and 9 females, mean age 29.7 ± 7.5 years old) and 15 healthy right-handed controls (8 males and 7 females, mean age 37.3 ± 8.5 years old). Healthy controls had no history of psychiatric or neurological diseases. All patients were screened independently by two psychiatrists to ensure that they met criteria for TRD. All fifteen patients were diagnosed with a major depressive episode of at least 2 years’ duration according to Diagnostic and Statistical Manual of Mental Disorders-Fourth Edition IV (DSM-IV) criteria, and all had a minimum score at entry of 35 (37.4 ± 3.0) on the 17-item Hamilton Depression Rating Scale (HDRS), indicating severe depression (27). A priori exclusion criteria were a) other psychiatric disorders with the exception of depression, such as schizophrenia, bipolar disorder, and obsessive–compulsive disorder; b) organic causes of depression including heart, liver, and kidney diseases; c) surgically implanted electronic devices or metal frame supporting equipment precluding MRI scanning.

Data set 1 was used to construct a probabilistic tractography map showing fibers directly connecting the PFC and sACC and the x-y-z coordinates of peak connectivity as the optimal site for TMS. Data set 2 was used for functional connectivity analysis to test the reliability of the optimal TMS site generated from the anatomic connectivity map.

Using probabilistic fiber tractography, we were able to infer anatomic connectivity that progressed into gray matter, and gained a comprehensive description of the connections between the sACC and PFC. By comparing the pattern of functional connectivity between TRD patients and healthy controls, we then verified the rationality of the TMS sites.

### Data Acquisition

All Data set 1 subjects were examined using a Signa HDx 3.0 Tesla MR scanner (General Electric, Milwaukee, WI, USA). The diffusion tensor imaging (DTI) scheme of Data set 1 yielded 64 images with non-collinear diffusion gradients (b=1000 s/mm^2^) and 3 non-diffusion-weighed images (b=0 s/mm^2^) using a single-shot echo planar imaging sequence (SE-EPI). An integrated parallel acquisition technique was used with an acceleration factor of 2 to reduce acquisition time and image distortion from susceptibility artifacts. From each participant, 75 slices were collected with FOV = 256×256 mm, acquisition matrix = 128×128, flip angle (FA) = 90°, and slice thickness = 2 mm with no gap. This method resulted in voxel-dimensions of 2×2×2 mm, TE = 67.6 ms, and TR = 8500 ms. Sagittal 3D T1-weighted images were also acquired with a brain volume (BRAVO) sequence (TR/TE= 8.1/3.1 ms, inversion time = 450 ms, FA = 13°, FOV = 256×256 mm, matrix = 128×128, slice thickness = 1 mm with no gap, 176 sagittal slices, and voxel size = 1×1×1 mm).

In Data set 2, imaging was performed using a Siemens 3.0-T MR system equipped with a SIEMENS MR HEADER coil. The protocol (ep2d.bold.REST.2000/30.4M.6MIN.FAST.0.49) included 64 phase encoding steps. The following acquisition parameters were used in the fMRI protocol: echo time = 30 ms, FOV = 256×256, field of view = 256 mm, acquisition matrix = 64×64, voxel size: 1×1×1 mm, slice thickness = 4 mm, no gap, 188 sagittal slices, TR = 2000 ms, TE = 30 ms, and FA = 90°.

### Data Preprocessing and Analysis of Data Set 1

Diffusion tensor and T1-weighted images from Data set 1 were visually inspected independently by two radiologists for obvious artifacts arising from subject motion and instrument malfunction. Distortions in diffusion-weighted images caused by eddy currents and simple head motions were corrected using the FMRIB Diffusion Toolbox (FSL) 4.0 (http://www.fmrib.ox.ac.uk/fsl). Skull-stripped T1-weighted images of each subject were co-registered to the subject’s non-diffusion-weighted image (b=0s/mm^2^) using the Statistical Parametric Mapping 8 (SPM8) package (http://www.fil.ion.ucl.ac.uk/spm), yielding a set of co-registered T1 images (rT1) in DTI space. Then the rT1 images were transformed to Montreal Neurological Institute (MNI) space. Seed masks and target masks were transformed from MNI space to native DTI space using nearest-neighbor interpolation.

Tractography was performed in diffusion space using the FSLpackage. Voxel-wise estimates of fiber orientation distribution were calculated using Bedpostx. Probability distributions for two fiber directions at each voxel were calculated using the multiple fiber extension (28) of a previously published diffusion modeling approach (29). The connection probability value was recorded for every seed voxel. We also transformed the connection probability map to MNI space and averaged the 20 individual connection probability maps to obtain a mean probability connectivity map for the seed. We then found the coordinate of maximum value in the mean probability connectivity map. Fiber tracking was used to obtain the fibers connecting the two areas. To map the anatomic connections between the PFC and sACC, these brain areas were defined in MNI space. The sACC was defined as that part of the ACC located beneath the genu of the corpus callosum and corresponds primarily to Brodmann’s area (BA)10 as well as the caudal portions of BA32 and BA24 (20). The sACC was drawn by hand based on BAs 10, 24, and 32. As the PFC as no clear anatomic boundary, we defined a liberal mask including middle frontal gyrus, superior frontal gyrus, and part of the orbitofrontal region to map the connections between sACC and PFC. The PFC was drawn on the structural template of MNI152. The inferior boundary was the inferior frontal sulcus, and the posterior boundary was the precentral sulcus.

### Data Preprocessing and Analysis of Data Set 2

We used Data set 2 to assess the accuracy of the anatomic connectivity derived from Data set 1 and to reveal differences in functional connectivity between TRD patients and healthy controls. First, DTI analysis of Data set 2 was used to demonstrate similar anatomical connectivity to normal subjects in Dataset 1. To test the rationality of the TMS sites identified by probabilistic tractography analysis of Data set 1, functional connectivity preprocessing was conducted using SPM8 Data Processing Assistant for Resting-State fMRI (DPARSF) V2.0 Advanced Edition (30) and Resting-State fMRI Data Analysis Toolkit (REST, http://www.restfmri.net). For statistical analysis, the contrast images of TRD patients and healthy controls were analyzed using a two-sample *t* test to compare the differences in functional connectivity between the right and left stimulation site.

## Results

**Figure 1:**
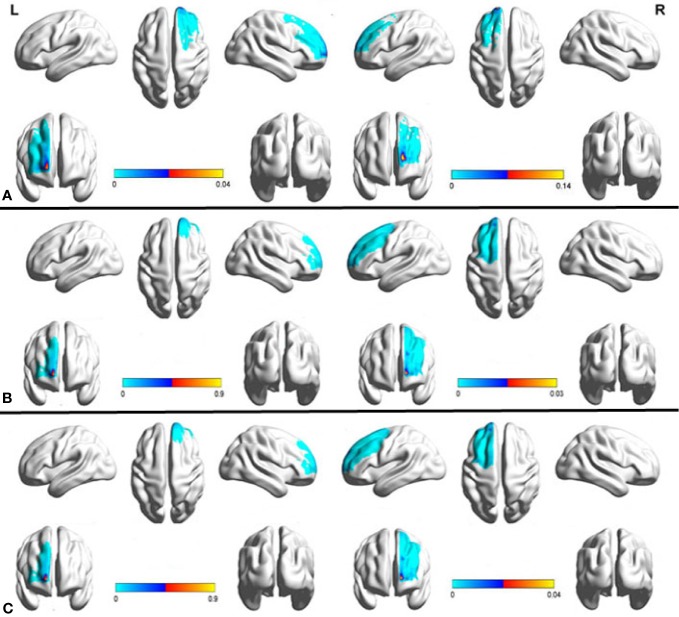
Mean probabilistic anatomic connectivity maps derived from healthy controls **(A, B)** and treatment-resistant depression (TRD) patients **(C)** using the subgenual anterior cingulate cortex (sACC) as the seed region. The connectivity map of each individual (a, n=20; b, n=15; c, n=15) was transformed to MNI space and averaged. The prefrontal cortex (PFC) coordinate of maximum value in the mean connectivity map was identified as the optimal site for transcranial magnetic stimulation (TMS) to modulate sACC activity. The results based on Data set 1 **(A)** were reproducible in Data set 2 **(B, C)**.

**Figure 2:**
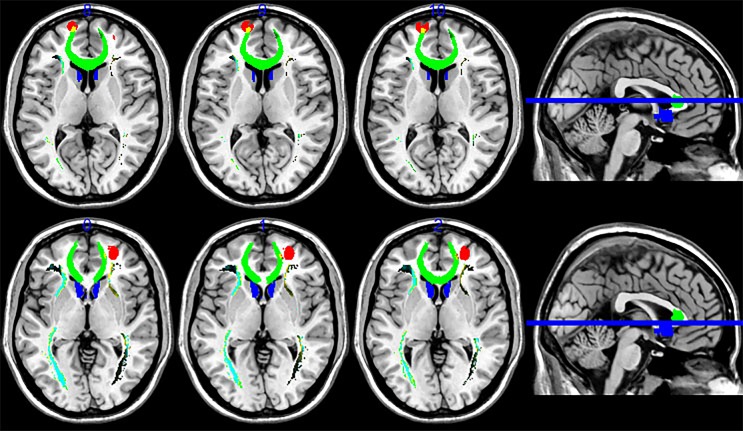
Fiber tractography map, including the prefrontal cortex (PFC) and sACC (seed region). PFC targets are in red and the sACC seed region is in blue, while fibers running through the seed region are in green. Fiber tracts connected mainly to the medial frontal areas, but also included a site of strong connectivity in the left PFC.

We used Data set 1 consisting of MRI scans from 20 healthy subjects to examine the anatomical connectivity (probabilistic tractography) between the sACC and PFC (**Supplementary Figure 1**). A probability connectivity map was derived for both hemispheres of each subject and mean probability connectivity maps obtained (**Figure 1**), yielded bilateral maximum probability coordination (x-y-z in MNI space) as potential TMS sites. Slight differences in connectivity were observed between left and right PFC, with the right maximum anterior to the left (left MNIxyz, −15, 65, 14; right MNIxyz, −15, 65, 5). Potential PFC stimulation sites were identified as voxels with the highest probability of connectivity to the sACC (**Supplementary Figure 2**). Fiber tracking from the sACC to PFC indicated that the inferior fronto-occipital fasciculus ran through the sACC and the left PFC probability peak (**Figure 2**).

To explore the reproducibility of this PFC–sACC connectivity, Data set 2 derived from another group of 15 healthy controls, as well as 15 TRD patients using a different MRI scanner was subjected to similar analysis. Again, the PFC region demonstrated a strong probability of anatomic connectivity with sACC. Further, while absolute values differed, the connectivity map of TRD patients was similar to that of the healthy controls (**Figure 1**), including near overlapping maximum value coordinates. Thus, our tractography method was highly reproducible among different populations.

**Figure 3:**
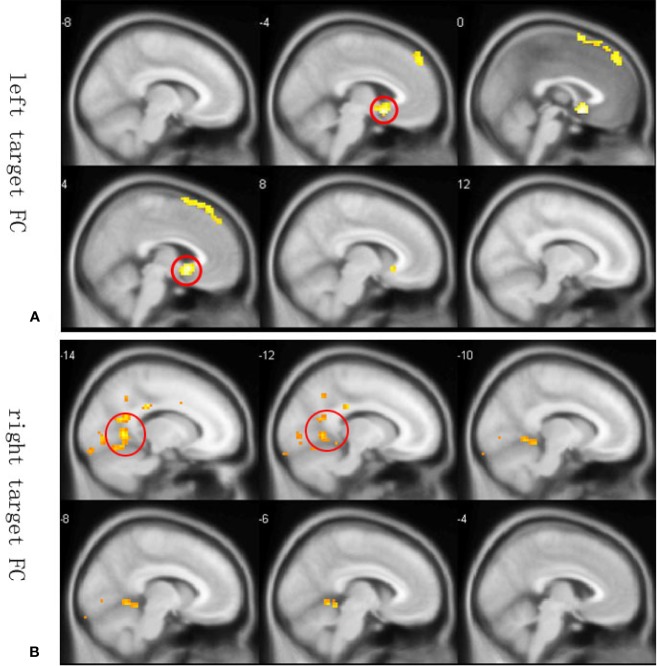
Whole-brain resting state functional connectivity differences between healthy controls and TRD patients. Functional connectivity ROIs were taken from our anatomic connectivity results (coordinates shown in **Figure 3**). **(A)** Functional connectivity between the left target and sACC (seed region for the anatomic connectivity, red circles in **A**) was significantly stronger in the healthy group. Only negative results are shown because a previous study reported that anticorrelation between the PFC and subgenual cingulate (BA25) is related to depression networks (22). **(B)** Functional connectivity with the right PFC target. Only negative results are shown. Red circles in Figure 3B illustrate PCC areas involved in depression (41).

Since resting state functional connectivity may differ from anatomical connectivity, we constructed whole-brain functional connectivity maps using the average MNI coordinates of maximum value in Data set 2 as ROIs. Peak functional connectivity of the left PFC with the left sACC was located exactly at the position of peak anatomic connectivity in both patients and controls, but was significantly stronger among healthy controls than TRD patients (**Figure 3A**). In contrast, the right PFC was functionally connected to the posterior cingulate cortex (PCC) (**Figure 3B**). Between-group comparisons across the whole brains of Data set 2 at an AlphaSim corrected *p <* 0.01 height threshold (*t* = 2.467, df = 28) confirmed reproducibility with anatomic connectivity analysis across disease states and MRI scanners (**Table 1**).

**Table 1:** Functional connectivity results in healthy controls versus depression patients.

L/R	Region	cluster	Peak MNI coordinate			peak Z score	Peak intensity
			x	y	z		
L	Limbic Lobe	60					
L	Anterior Cingulate	54	0	9	-12	3.62	-4.30
L	Brodmann area 25	30					
R	Limbic Lobe	25					
R	Cerebelum_4_5_L (aal)	20					
R	Cerebelum_6_L (aal)	20	-15	-57	0	3.89	-4.30
R	Calcarine_L (aal)	16					
R	Posterior Cingulate	14					

Two sample t-test, Alphasim corrected, p < 0.01, cluster size = 19(left), cluster size = 11(right).

## Discussion

In this study, we identified PFC sites with strong anatomical connectivity to the sACC, and PCC that may be optimal targets from TMS treatment of depression. Further, these sites were reproducible across different data sets and closely mirrored functional connectivity. In light of the strong evidence that modulation of sACC and PCC activity can mitigate symptoms of depression, we suggest that these PFC sites are optimal targets for TMS treatment.

Depression is linked to reduced activity of the left PFC (26, 31, 32). Recently, Wang et al. (33) reported that the effective connectivity from DLPFC to right angular gyrus predicts the antidepressant effects of electroconvulsive therapy (ECT) for MDD (33). Further, a recent functional connectivity study found that dysfunctional communication between the medial PFC and right ventral anterior insula is a major contributor to MDD pathogenesis (34). The application of repetitive (r)TMS for depression was initially driven by functional imaging data showing reduced left PFC activity in depression (35, 36). Based on empirical experience, the majority of the initial studies applied high-frequency rTMS to the left PFC (13, 25, 37, 38). However, low-frequency stimulation of the right PFC has also been used (17, 39) with clinically significant efficacy (37, 40). Grimm et al. (41) found that depressed patients exhibited hypoactivity in the left PFC during both unattended and attended emotional judgments but hyperactivity in the right PFC during attended emotional judgment (41), suggesting distinct forms of dysfunction in left and right PFC–ACC pathways Our current study also provides evidence that sites within both right and left PFC are strongly connected to regions involved in TRD, consistent with previous studies(13, 22, 23), and so may be effective TMS targets.

Repetitive TMS applied at a variety of sites and frequencies has proven highly effective for inducing mood changes in healthy controls and therapeutic effects in TRD patients. For instance, concomitant high-frequency rTMS stimulation of the left PFC and low-frequency rTMS of the right PFC was reported to have antidepressive effects. Alternatively, these regimens were ineffective when the stimulation side was reversed (37, 40, 42), suggesting an imbalance in frontal lobe activity (hypoactivity in the left frontal lobe and excessive inhibitory activity in the right frontal lobe) among MDD patients. Left and right PFC sites with strongest anatomic connectivity also appear to have non-overlapping functional targets, the sACC (left target) and PCC (right target), both of which are involved in depression (41). Therefore, analyzing the connectivity of sACC (43) and PCC (44) could reveal the mechanisms underlying therapeutic responses to TMS at these sites. Our study provides further evidence that the left PFC connects directly to the sACC and the right PFC connects to the PCC, consistent with previous studies (22, 43, 44). Therefore, this study suggests that the antidepressant mechanisms of TMS are mediated by modulation of left PFC–sACC neural circuit activity and right PFC–PCC activity.

Multiple targets have been used for rTMS treatment (26) as there is no current consensus on the optimal target for antidepressant efficacy. Tracing direct fiber pathways from deep regions implicated in depression, such as the ACC, to cortical areas accessible to modulation by TMS is a novel approach for targeting. We performed probabilistic tractography in healthy controls and TRD patients to determine the connectivity patterns of the sACC and PFC. The subcallosal cingulate is connected to many ipsilateral regions, such as the medial frontal cortex, ACC, and PCC (4), but these regions have limited area for TMS. Here we identified strong connection with specific sites of the left and right PFC that may be superior targets for treating TRD. The left PFC site is located on the gyrus, while the right PFC target is located on the sulcus, so the left PFC target may be more readily activated by TMS. Nonetheless, stimulation of both sides with distinct frequency patterns may provide the best therapeutic effect (6, 37, 42–44).

The DLPFC position for TMS is often located using the electroencephalography (EEG)-based “5-cm rule” or by neuroimaging-based targeting of BA9 or BA46 (42, 45–47). While the 5-cm rule is an easy low-cost option, it does not account for individual differences in skull size. Further, targeting based on BA9 or BA46 locations on neuroimages did not substantially improve clinical efficacy compared to the 5-cm rule (22). We suggest that the optimal target can be identified using a TMS Navigation system and our MNI coordinates.

This study has several limitations. First, only 15 TRD patients were examined and MDD is a heterogeneous disorder, so certain abnormalities in anatomic or functional connectivity may have been missed. Second, the sACC subregions connected anatomically with the PFC were not examined.

## Conclusions

This study identifies sites in right and left prefrontal cortex that may be optimal targets for transcranial magnetic stimulation treatment of depression based on strong functional connectivity with the posterior and subgenual anterior cingulate cortex.

## Data Availability Statement

The datasets generated for this study are available on request to the corresponding authors.

## Ethics Statement

The studies involving human participants were reviewed and approved by The Ethical Committee of the First Affiliated Hospital of Jilin University. The patients/participants provided their written informed consent to participate in this study. Written informed consent was obtained from the individual(s) for the publication of any potentially identifiable images or data included in this article.

## Author Contributions

TJ and LL designed the study. SL, LZ, HZ, and YY collected the samples and participants’ characteristics. YY, QT, HY, and LF analyzed and discussed the experimental results. QT, YY, XS, and TJ wrote the paper. All authors contributed to and have approved the final manuscript.

## Funding

This work was supported by the National Natural Science Foundation of China (81671330 to LL, 81571318 to XS); Medical Science and Technology Research Project of Henan Province (2018020373 to HY); Health and Family Planning Commission of Henan Province (201501015 to XS), Department of Science and Technology of Henan Province (162102410061 to XS; No. 2017JQ023 to XS), and School and Hospital Co-incubation Funds (2017-BSTDJJ-04 to XS) and Research Council of Norway (223273).

## Conflict of Interest

The authors declare that the research was conducted in the absence of any commercial or financial relationships that could be construed as a potential conflict of interest.

## Supplementary Material

Supplementary Figure 1 Regions of interest (ROIs) for connectivity analysis. (a) Left prefrontal cortex (black) and right prefrontal cortex (yellow) ROIs. (b) The seed region (sACC, red) contains effective therapeutic target for DBS and VNS (7, 19).

Supplementary Figure 2 Optimal TMS sites were defined as the coordinates of maximum values in the probability connectivity map (left MNI x-y-z -15, 65,14; right MNI x-y-z 24, 53, 1). The radius was 8 mm. These targets were also used as seed ROIs to conduct functional connectivity analysis.
