# Supplementary figures and images for: Anatomical Connectivity-Based Strategy for Targeting Transcranial Magnetic Stimulation as Antidepressant Therapy
Source: Front Psychiatry. 2020 Apr 3;11:236. doi: 10.3389/fpsyt.2020.00236 (PMC7145890; doi:10.3389/fpsyt.2020.00236)

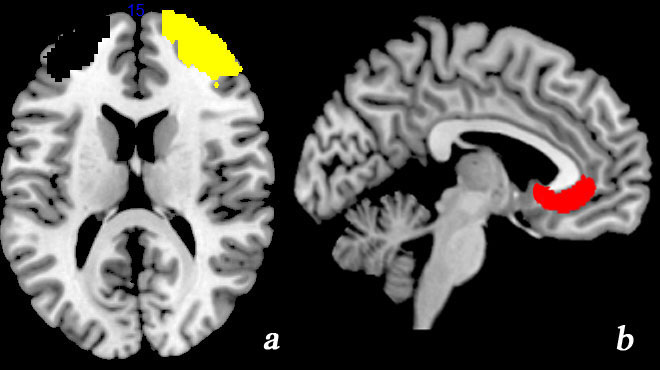

Supplement: Supplementary Figure 1 — Regions of interest (ROIs) for connectivity analysis. (a) Left prefrontal cortex (black) and right prefrontal cortex (yellow) ROIs. (b) The seed region (sACC, red) contains effective therapeutic target for DBS and VNS (7, 19). [file Image_1.tif]

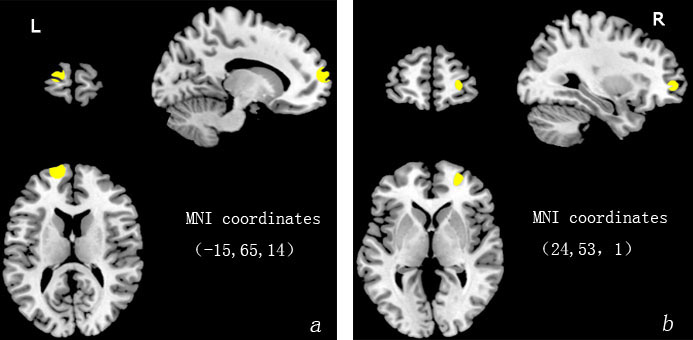

Supplement: Supplementary Figure 2 — Optimal TMS sites were defined as the coordinates of maximum values in the probability connectivity map (left MNI x-y-z -15, 65,14; right MNI x-y-z 24, 53, 1). The radius was 8 mm. These targets were also used as seed ROIs to conduct functional connectivity analysis. [file Image_2.tif]
